# Low SARS-CoV-2 seroprevalence but high perception of risk among healthcare workers at children’s hospital before second pandemic wave in Germany

**DOI:** 10.1007/s12519-021-00447-8

**Published:** 2021-08-20

**Authors:** Marietta Neumann, Annette Aigner, Eileen Rossow, David Schwarz, Maria Marschallek, Jörg Steinmann, Ralf Stücker, Ingo Koenigs, Philippe Stock

**Affiliations:** 1 Department of Paediatrics, Altona Children’s Hospital, Universität Hamburg, Altonaer Kinderkrankenhaus, Bleickenallee 38, 22763 Hamburg, Germany; 2 Corporate Member of Freie Universität Berlin and Humboldt-Universität Zu Berlin, Institute of Biometry and Clinical Epidemiology Berlin, Charité–Universitätsmedizin Berlin, Berlin, Germany; 3 Department of Neonatology and Paediatric Intensive Care Medicine, Altona Children’s Hospital, Hamburg, Germany; 4 Department of Paediatric Surgery, Altona Children’s Hospital, Hamburg, Germany; 5 Labor Dr. Fenner and Colleagues, Hamburg, Germany; 6 Department of Paediatric Orthopaedics, Altona Children’s Hospital, Hamburg, Germany; 7 Department of Paediatric Surgery, University Medical Centre Hamburg-Eppendorf (UKE), Hamburg, Germany; 8 Department of Paediatrics, Altona Children’s Hospital, Hamburg, Germany

**Keywords:** Health personnel, SARS-CoV-2, Seroepidemiologic studies, Pediatrics

## Abstract

**Background:**

Healthcare workers are considered a particularly high-risk group during the coronavirus disease 2019 (COVID-19) pandemic. Healthcare workers in paediatrics are a unique subgroup: they come into frequent contact with children, who often experience few or no symptoms when infected with severe acute respiratory syndrome coronavirus 2 (SARS-CoV-2) and, therefore, may transmit the disease to unprotected staff. In Germany, no studies exist evaluating the risk of COVID-19 to healthcare workers in paediatric institutions.

**Methods:**

We tested the staff at a large children’s hospital in Germany for immunoglobulin (Ig) G antibodies against the nucleocapsid protein of SARS-CoV-2 in a period between the first and second epidemic wave in Germany. We used a questionnaire to assess each individual’s exposure risk and his/her own perception of having already been infected with SARS-CoV-2.

**Results:**

We recruited 619 participants from all sectors, clinical and non-clinical, constituting 70% of the entire staff. The seroprevalence of SARS-CoV-2 antibodies was 0.325% (95% confidence interval 0.039–1.168). Self-perceived risk of a previous SARS-CoV-2 infection decreased with age (odds ratio, 0.81; 95% confidence interval, 0.70–0.93). Having experienced symptoms more than doubled the odds of a high self-perceived risk (odds ratio, 2.18; 95% confidence interval, 1.59–3.00). There was no significant difference in self-perceived risk between men and women.

**Conclusions:**

Seroprevalence was low among healthcare workers at a large children’s hospital in Germany before the second epidemic wave, and it was far from a level that confers herd immunity. Self-perceived risk of infection is often overestimated.

## Introduction

The COVID-19 pandemic has caused a burden to individuals, the healthcare system, and economies worldwide. The role of children in the spread of the pandemic remains unclear and is subject to ongoing research and frequent discussion at the political level. Transmission patterns in schools, childcare centres and households have been the basis of most epidemiologic studies involving children and COVID-19 [1–4], but to our knowledge, no studies exist assessing whether adults in close contact with children in their work environment are more likely to acquire the disease. However, evidence concerning the course of COVID-19 in children has been consistent: it is often mild or without any symptoms at all [5–8]. Seroprevalence may be up to sixfold higher than reported cases in children, implying large numbers of undiagnosed infected children [9].

Healthcare workers (HCWs) are considered a particularly high-risk group in the course of any infectious epidemic. In the 2009 influenza pandemic, HCWs were disproportionately affected [10], and the same appears to be true for COVID-19 [11]. However, research has also yielded heterogeneous results, suggesting a variety of risk and protective factors. A single study in Spain found a low seroprevalence among paediatric HCWs [12], but other than that, little is known about this subgroup despite the uncertainty of SARS-CoV-2 transmissibility by children.

To help close this gap, we tested staff members at a large children’s hospital in Germany for SARS-CoV-2 immunoglobulin (Ig) G antibodies, to quantify the previous exposure to SARS-CoV-2 among this particular subpopulation. We hypothesized that HCWs at a children’s hospital might be at a particularly high risk of SARS-CoV-2 exposure because children with asymptomatic COVID-19 present for unrelated reasons, and, therefore, HCWs come into unsuspected close contact without adequate personal protective equipment (PPE). To our knowledge, this is the first seroprevalence study for paediatric HCWs in Germany.

In addition to the potentially measurable risk for an infection, the self-perceived risk of an individual due to his/her everyday exposures is of great scientific interest. It may be an expression of how serious the threat by this ongoing pandemic is considered, and of the confidence placed in strategies applied to limit it. It may also influence preventive behaviours, such as social distancing and handwashing [13]. A nationwide survey in Germany found that 60% of HCWs had concerns regarding their own health [14], and Behrens et al. found that while actual seroprevalence was low, the perceived risk among HCWs at a German hospital was considerable and decreased with time. They also found that women had a higher self-perceived risk than men [15]. Understanding risk perceptions in the general public and among subgroups may ultimately guide effective communication about the pandemic to the public.

## Methods

### Ethical considerations

This study was approved by the Ethical Review Committee of the Medical Association (Aerztekammer) Hamburg in Germany (PV7404). Written informed consent was obtained from all participants prior to participation in the study.

### Recruitment

**Fig. 1:**
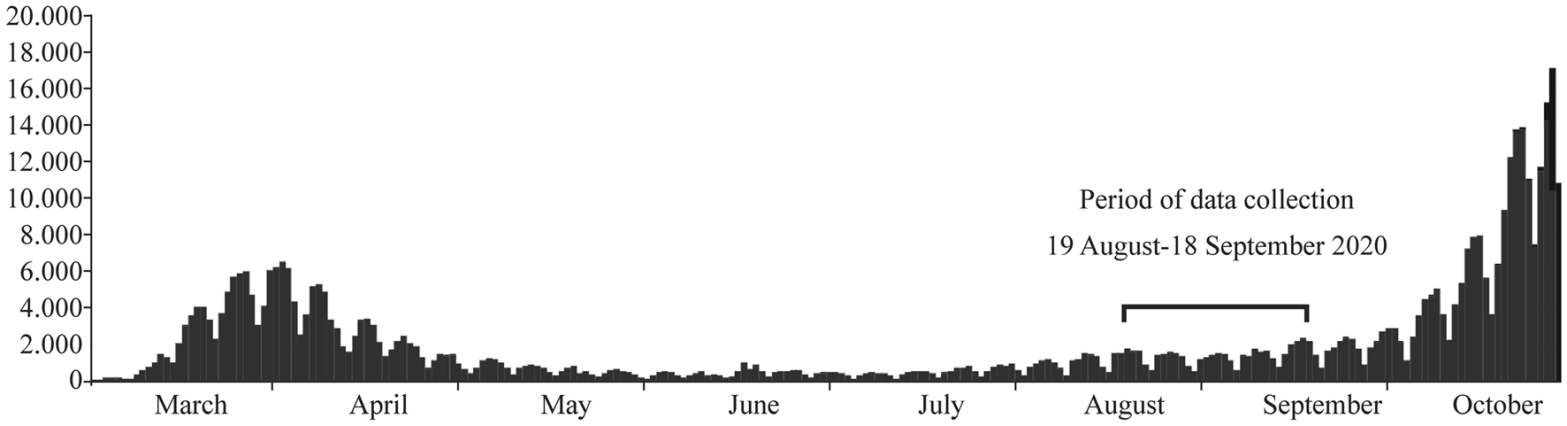
New SARS-CoV-2 infections per day in Germany (*y*-axis) in the time period from March to October 2020 (*x*-axis). Indicated is the period during which data were collected for this study. (This figure was adapted from: https://experience.arcgis.com/experience/478220a4c454480e823b17327b2bf1d4)

We aimed at recruiting all personnel working at the Altona Children’s Hospital in Hamburg, Germany, which constitutes a deliberately chosen cluster of the entire population of paediatric hospital staff; therefore, we used non-probability cluster sampling with subsequent total population sampling. Staff members were recruited with announcements on internal mailing lists and were additionally addressed personally. Staff members across all sectors were invited to participate, including trainees and non-clinical staff. Non-clinical HCWs, while presumably at a lower exposure risk, were included to diversify our sample. We excluded individuals with congenital or acquired immunodeficiencies, those who had been absent from work since before COVID-19 emerged in Germany, and minors under the age of 18 years. Data were acquired over a 1-month period, 6 months after the first COVID-19 case in Hamburg was diagnosed, i.e. after the first epidemic wave (Fig. 1).

### Test methods

Serum blood samples were obtained from each subject and were tested for SARS-CoV-2 anti-nucleocapsid IgG antibodies by enzyme-linked immunosorbent assay (ELISA) using a CE-marked kit ‘Anti-SARS-CoV-2 NCP Elisa (IgG)’ by EUROIMMUN, Lübeck, Germany. This is a semi-quantitative assay based on the ratio of the extinction of the sample and that of a calibrator. A ratio of < 0.8 is considered a negative result, ≥ 1.1 positive, and ≥ 0.8 and < 1.1 borderline. EUROIMMUN reports a test sensitivity of 94.6% when samples are obtained more than 10 days after symptom onset, and a specificity of 99.8%. The rationale for using this assay was the superior immunogenicity of the nucleocapsid structural protein (N) compared to the spike (S) antigens in SARS-CoV [16]. Anti-N antibody tests in SARS-CoV-2 are detected earlier and are more sensitive than anti-S antibodies [17].

Positive and borderline results were further tested for anti-S1 IgG antibodies using a CE-marked and FDA-approved kit “Anti-SARS-CoV-2 Elisa (IgG)” by EUROIMMUN. This assay tests for the presence of antibodies against the S1 structural protein, including antibodies against the receptor-binding domain of S1. Interpretation is identical to the above-mentioned anti-N ELISA. This assay has been externally validated [18–20]. The manufacturer reports a sensitivity of 80% if measured > 10 days after symptom onset, and a specificity of 99.6%. We included this second test based on the correlation between the kinetics of neutralising antibodies and those of antibodies targeting S1 and the receptor-binding domain [17, 21], to establish whether seropositive individuals were also likely to be immune to infection.

Prior to sample collection, each subject was asked to complete a short questionnaire to assess his/her individual exposure risk based on job category, time spent at work, known exposures with or without protective gear, symptoms of respiratory illness, and travel to a high-risk area. SARS-CoV-2 exposures were classified according to contact categories established by the Robert Koch Institute [22] to guide post-exposure measures [22]. “Full PPE” was specified in the questionnaire as constituting an FFP2 face mask, eye shield or goggles, gloves, and gown.

Furthermore, participants were asked how high they perceived their own risk of having previously been infected with SARS-CoV-2, as previously done by Behrens et al. at another German hospital [15]. The relevant survey question was “How high do you estimate the chance (in %) of having been infected with the novel coronavirus already?”, indicating 0% as no chance at all, and 100% as completely certain. We divided answers into groups of “very low” (< 1%), “low” (1 to < 20%), “medium” (20% to < 50%), “high” (50%), and “very high” (> 50%).

### Statistical analysis

We report absolute and relative frequencies for categorical variables, median along with interquartile range (IQR) for ordinal and continuous variables—for the whole cohort, just as by test result. Prevalence estimates of positive tests are reported along with 95% confidence intervals (CIs) based on Clopper and Pearson [23]. Risk factors for a higher self-perceived risk were investigated with ordinal logistic regression models, results displayed as odds ratio (OR) estimates along with 95% CIs. Because only 6.7% of observations for the relevant variables were missing, a complete-case analysis was carried out. Statistical analyses were performed using R [24], along with additional *R* packages for data handling and plotting [25].

## Results

Between 19 August and 18 September 2020, 619 staff members were recruited, constituting 70.3% of total staff. The response rate varied among the different professions between 51.2% (service staff) and 87.3% (administrative staff).

Of the 616 participants who completed the questionnaire, 518 (84.0%) were female, and the median age was 38.5 years (IQR = 29.00, 50.00). Nurses made up the majority of participants (46.0%), followed by physicians (19.9%), and administrative staff (8.6%). Physicians were on average slightly younger than nurses and much older than trainees (median IQR: physicians: 38.0 (32.5, 45.0), nurses: 41.0 (30.0, 52.0), trainees: 22.0 (20.0, 24.3)). The majority of participants (84.2%) had been hired prior to the outbreak of the pandemic in Germany. Symptoms of a respiratory tract infection at any time since the beginning of the outbreak in Germany were reported by 245 participants (39.9%), and 75 (12.2%) had been to areas or countries considered high risk for SARS-CoV-2 transmission at that time by the German institute for disease control and prevention [26]. 170 participants (27.7%) had previously been tested for SARS-CoV-2 RNA by real-time polymerase chain reaction (RT-PCR) on nasopharyngeal throat swabs in March or April 2020, out of whom 4 (0.7%) had a positive result.

Previous contact with a person with a known SARS-CoV-2 infection at work was reported by 64 participants (10.4%), and 41 (64.1%) of these had occurred with full PPE, 13 without PPE and at < 2 m distance for more than 15 min, and 10 without PPE and contact > 2 m distance. Table 1 details the characteristics of the study population by test results.

**Table 1:** Characteristics of the study population by test results

Variables	Negative test result (*n* = 607)	Positive test result (*n* = 2)	Borderline test result (*n* = 7)	Total (*n* = 616)
Self-perceived risk				
Very low (< 1%)	139 (23.8%)	0 (0.0%)	2 (28.6%)	141 (23.8%)
Low (1 to < 20%)	150 (25.7%)	0 (0.0%)	2 (28.6%)	152 (25.6%)
Medium (20 to < 50%)	138 (23.6%)	0 (0.0%)	1 (14.3%)	139 (23.4%)
High (50%)	129 (22.1%)	1 (50.0%)	1 (14.3%)	131 (22.1%)
Very high (> 50%)	28 (4.8%)	1 (50.0%)	1 (14.3%)	30 (5.1%)
Missing	23 (3.79%)	0 (0%)	0 (0%)	23 (3.73%)
Age				
Median (IQR)	39.00 (29.00, 50.00)	31.00 (26.50, 35.50)	38.00 (25.00, 46.00)	38.50 (29.00, 50.00)
Sex				
Female	511 (84.5%)	2 (100.0%)	7 (100.0%)	520 (84.7%)
Male	94 (15.5%)	0 (0.0%)	0 (0.0%)	94 (15.3%)
Missing	2 (0.33%)	0 (0%)	0 (0%)	2 (0.32%)
Professional group				
Physicians	122 (20.1%)	0 (0.0%)	1 (14.3%)	123 (20.0%)
Service staff	42 (6.9%)	0 (0.0%)	0 (0.0%)	42 (6.8%)
Trainees	71 (11.7%)	0 (0.0%)	1 (14.3%)	72 (11.7%)
Allied health staff	38 (6.3%)	0 (0.0%)	1 (14.3%)	39 (6.3%)
Nurses	279 (46.0%)	2 (100.0%)	4 (57.1%)	285 (46.3%)
Administrative staff	55 (9.1%)	0 (0.0%)	0 (0.0%)	55 (8.9%)
Date of employment				
Before 27/1/20	515 (85.1%)	1 (50.0%)	6 (85.7%)	522 (85.0%)
After 27/1/20	90 (14.9%)	1 (50.0%)	1 (14.3%)	92 (15.0%)
Missing	2 (0.33%)	0 (0%)	0 (0%)	2 (0.32%)
Part-time/full-time employment				
≤ 50%	99 (16.6%)	1 (50.0%)	2 (28.6%)	102 (16.9%)
51–75%	128 (21.5%)	0 (0.0%)	0 (0.0%)	128 (21.2%)
76–100%	369 (61.9%)	1 (50.0%)	5 (71.4%)	375 (62.0%)
Missing	11 (1.81%)	0 (0%)	0 (0%)	11 (1.79%)
Previously tested by RT-PCR				
No previous PCR	436 (73.6%)	1 (50.0%)	3 (42.9%)	440 (73.2%)
PCR negative	153 (25.8%)	0 (0.0%)	4 (57.1%)	157 (26.1%)
PCR positive	3 (0.5%)	1 (50.0%)	0 (0.0%)	4 (0.7%)
Missing	15 (2.47%)	0 (0%)	0 (0%)	15 (2.44%)
COVID-19 contact outside of work				
Yes	18 (3.0%)	0 (0.0%)	0 (0.0%)	18 (3.0%)
Missing	6 (0.99%)	0 (0%)	0 (0%)	6 (0.97%)
COVID-19 contact at work				
< 2 m away with PPE	39 (6.6%)	0 (0.0%)	2 (28.6%)	41 (6.8%)
< 2 m away without PPE	13 (2.2%)	0 (0.0%)	0 (0.0%)	13 (2.2%)
> 2 m away without PPE	9 (1.5%)	0 (0.0%)	1 (14.3%)	10 (1.7%)
None	492 (83.0%)	2 (100.0%)	4 (57.1%)	498 (82.7%)
No contact with patients at all	44 (7.4%)	0 (0.0%)	0 (0.0%)	44 (7.3%)
Missing	14 (2.31%)	0 (0%)	0 (0%)	14 (2.27%)
Previous symptoms of respiratory tract infection				
Yes	238 (39.9%)	2 (100.0%)	4 (57.1%)	244 (40.3%)
Missing	10 (1.65%)	0 (0%)	0 (0%)	10 (1.62%)
Travel to high risk area				
Yes	74 (12.3%)	0 (0.0%)	1 (14.3%)	75 (12.3%)
Missing	7 (1.15%)	0 (0%)	0 (0%)	7 (1.14%)
If travel to high risk area, country visited:				
Egypt	1 (1.4%)	0	0 (0.0%)	1 (1.4%)
France	1 (1.4%)	0	0 (0.0%)	1 (1.4%)
Italy	12 (16.7%)	0	0 (0.0%)	12 (16.4%)
Austria	45 (62.5%)	0	1 (100.0%)	46 (63.0%)
Switzerland	1 (1.4%)	0	0 (0.0%)	1 (1.4%)
Spain	9 (12.5%)	0	0 (0.0%)	9 (12.3%)
USA	3 (4.2%)	0	0 (0.0%)	3 (4.1%)

*IQR* interquartile range, *RT-PCR* real-time polymerase chain reaction

### Central research question

Of the 619 screened participants, two had a positive serology for SARS-CoV-2 nucleocapsid IgG antibodies. This implies a prevalence of 0.325%, with a 95% CI of 0.039–1.168. The characteristics of the two positively tested participants are displayed in detail in Table 2. All borderline test results for anti-N antibodies had negative serologies for anti-S1 antibodies.

**Table 2:** Characteristics of the two participants who tested positive for anti-N antibodies

Variables	Participant 1	Participant 2
Age	40	22
Sex	Female	Female
Profession	Nurse	Nurse
Part-time vs full-time	Part-time (≤ 50%)	Full-time
Contact outside of work	No	No
Contact at work	No	No
Symptoms	Yes	Yes
Ever tested for SARS-CoV-2 by RT-PCR	No	Yes, positive
Travel to high risk area	No	No
Self-reported risk (%)	50%	100%
Presence of anti-S1 antibodies	No	Yes

*RT-PCR* real-time polymerase chain reaction, *anti-S* anti-spike 1

### Self-perceived risk of infection

**Fig. 2:**
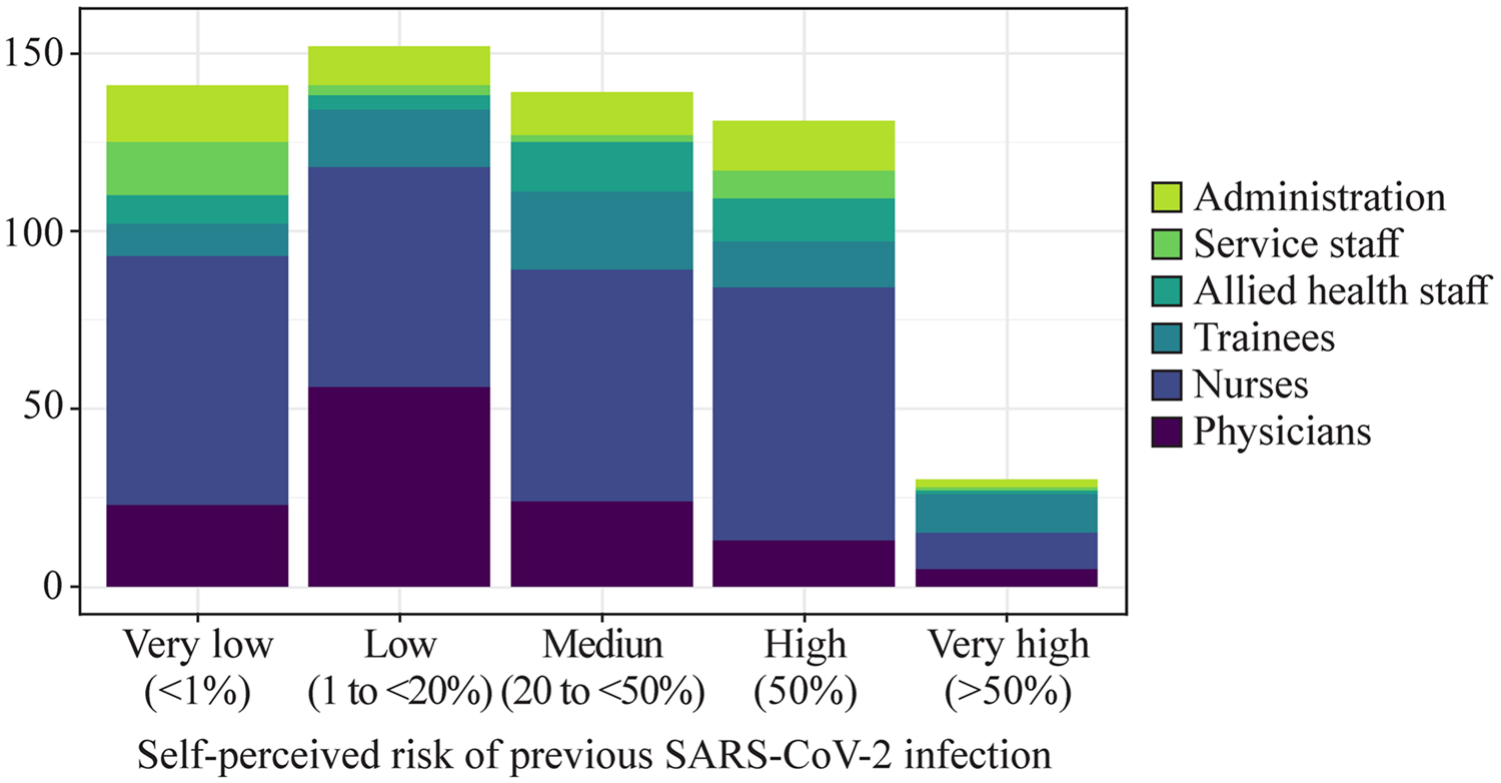
Distribution of professional groups across categories of their self-perceived risk of having previously been infected with SARS-CoV-2

We categorised answers into groups of “very low” (< 1%), “low” (1 to < 20%), “medium” (20% to < 50%), “high” (50%), and “very high” (> 50%) self-perceived risk, as illustrated in Fig. 2 by professional groups.

**Fig. 3:**
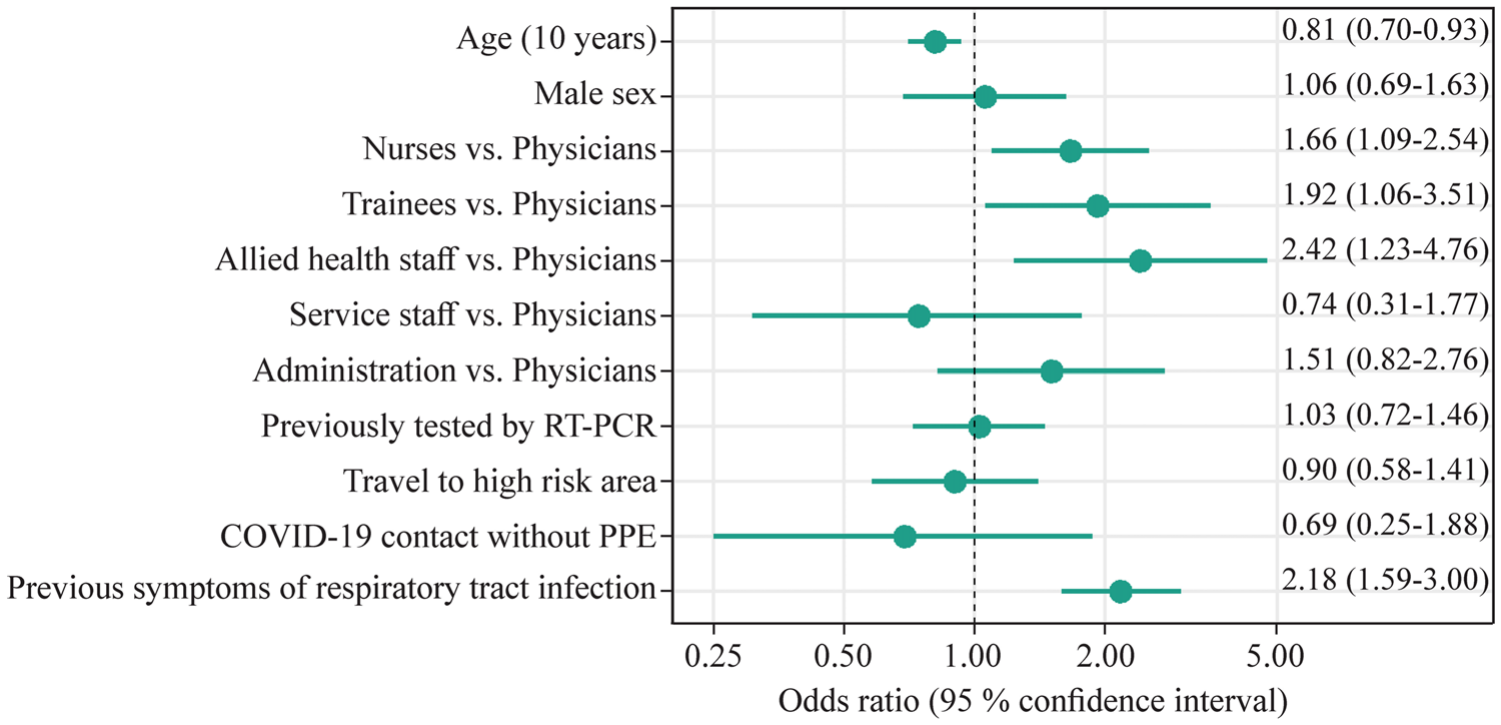
Association of different characteristics with the self-perceived risk of infection. An odds ratio estimate > 1 indicates that self-perceived risk increased when the respective characteristic was present and vice versa. The association with age is displayed in 10-year increments. Physicians were used as reference for odds ratios within professions. *RT-PCR* real-time polymerase chain reaction, *PPE* personal protective equipment

Self-perceived risk decreased with age, where an increase in age by 10 years reduced the odds of a higher self-perceived risk by 19% (OR, 0.81; 95% CI, 0.70–0.93). Having had symptoms more than doubled the odds of a high self-perceived risk (OR, 2.18; 95% CI, 1.59–3.00). Additionally, the profession was associated with the self-perceived risk of previous infection, where compared to physicians all other professional groups had higher odds of a high self-perceived risk. The odds of higher self-perceived risk were more than two-fold for allied health staff, almost two-fold for trainees, and 1.7-fold for nurses, compared to physicians (Fig. 3). There was no significant difference in self-perceived risk between men and women, given adjustments for the other characteristics.

## Discussion

After the first epidemic wave of COVID-19 in Germany, the seroprevalence of SARS-CoV-2-specific antibodies at Altona Children’s Hospital was low at 0.325% (95% CI 0.039–1.168). Self-perceived risk of a previous infection varied widely among subgroups within our sample but was consistently higher than the actual risk.

### Risk of COVID-19 infection in healthcare workers in general and in our study

**Fig. 4:**
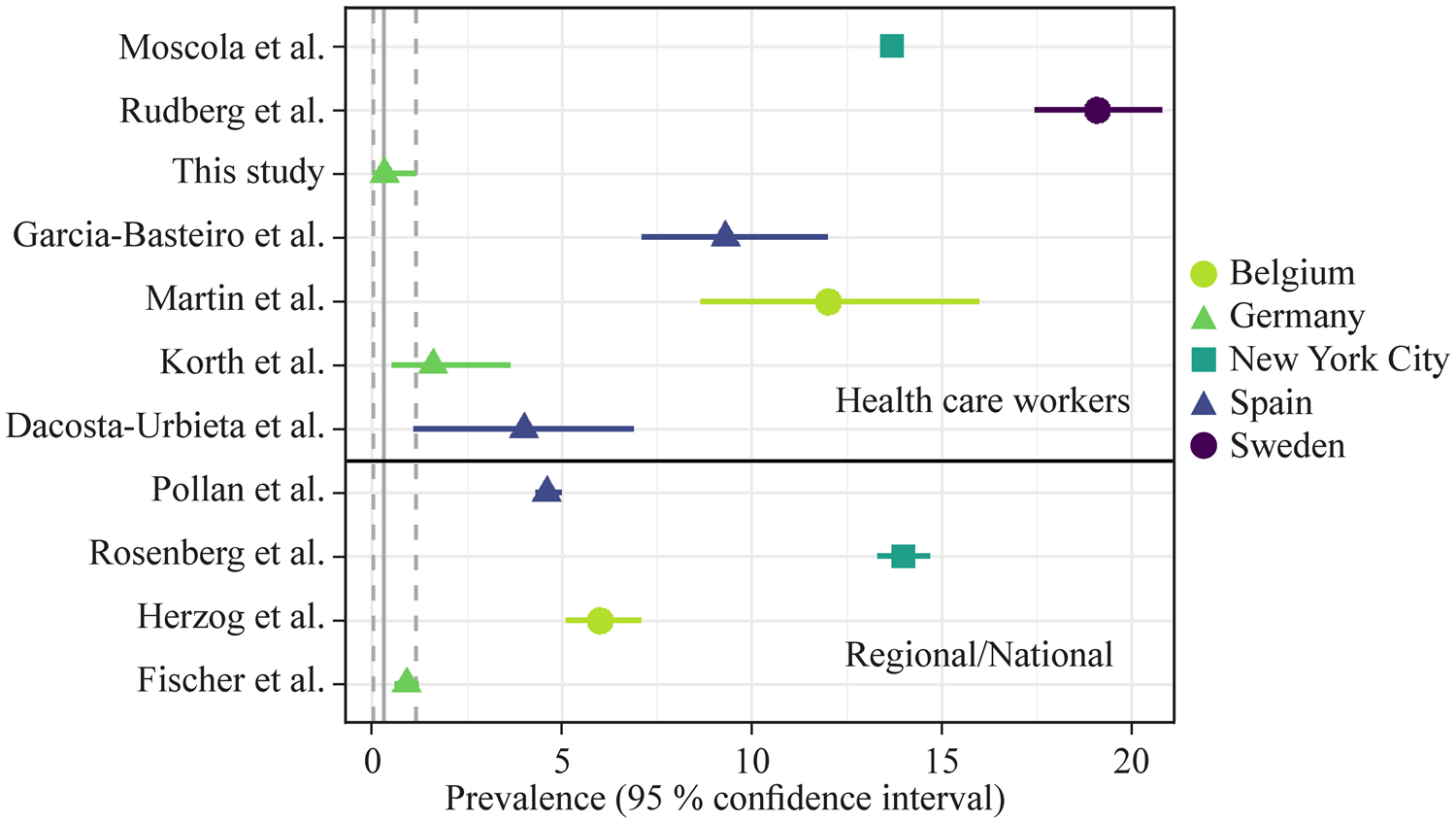
Comparison of seroprevalence findings among healthcare workers with regional or national levels in various countries. This figure used data from Moscola et al. [33], Rudberg et al. [31], Garcia-Basteiro et al. [30], Martin et al. [34], Korth et al. [32], Dacosta-Urbieta et al. [12], Pollán et al.[35], Rosenberg et al.[36], Herzog et al.[37], Fischer et al.[38]

HCWs are a particularly high-risk group for contracting SARS-CoV-2 [11], but research has also yielded heterogeneous results. Early on during the pandemic, when adequate personal protective equipment was not routinely used, doctors exposed to COVID-19 patients had a higher seroprevalence of SARS-CoV-2-specific antibodies than their unexposed colleagues [27]. Two later studies testing HCWs by RT-PCR from nasopharyngeal swabs found infection rates similar to community incidence and found no additional exposure risk for HCWs [28, 29]. However, seroprevalences at two large hospitals in high-burden areas in Spain and Sweden were higher than the estimated average in that region [30, 31]. Yet other investigators found surprisingly low seroprevalences among HCWs, including those specifically caring for COVID-19 patients at work [12, 32] (Fig. 4).

Several factors may be responsible for this variation. PPE shortage and inconsistent use of PPE were associated with a higher SARS-CoV-2 seroprevalence in a US-American multi-centre study [39]. During the early stage of the pandemic in China, a high seroprevalence was linked to insufficient PPE use [27]. Further, there is an apparent association between community incidence and HCW seroprevalence in that community [39]. In Germany and in several other countries, the epidemic occurred in waves with defined peaks and troughs. Timing of testing in the context of these epidemic waves influences results [34] and may lead to seemingly discordant results between different studies in a similar population. Finally, sensitivities and specificities vary between different test kits and poorer quality tests may lead to relevant measurement error, particularly in small sample studies.

### Possible reasons for low seroprevalence measured in our study population

Our study yielded a low seroprevalence compared to other German hospitals [15, 32], to a children’s hospital in Spain [12], and to the German general public [38], although the difference to the general public was not significant. There may be several reasons for this: (1) low case load: although local SARS-CoV-2 incidence in Hamburg was consistently above the national average [40], few patients with known COVID-19 had presented to our hospital by the time we completed data collection: Out of 19,539 children and accompanying adults who presented to our hospital between 27 January and 18 September, 8,067 were tested for SARS-CoV-2 by RT-PCR. Of these, only 13 were positive (8 inpatients or inpatients’ caregivers, and 5 outpatients); (2) Universal testing: starting from 12 May 2020, every admitted patient and accompanying adult caregiver was tested for SARS-CoV-2 regardless of symptoms, further reducing the risk of inadvertent exposure; (3) PPE use: Guidelines for adequate PPE use were instituted early on at Altona Children’s Hospital, soon followed by mandatory infection prevention measures including use of a surgical mask at all times by staff, by patients above the age of 6 years, and by caregivers. As the pandemic peak in Germany occurred later than in several other countries, there was more time to prepare and to acquire resources. Overall, few SARS-CoV-2 contacts (64) were reported by study participants, and the majority of these (64.1%) had occurred with full PPE. (4) Antibody dynamics: importantly, there may have also been a decline in antibody titres leading to a negative result in participants who had previously been infected nonetheless. This decline has been documented in follow-up studies of convalescent COVID-19 patients, particularly in asymptomatic individuals [41–43]. A study of a large community sample in England demonstrated a decline in overall seroprevalence over time [44]. Also, asymptomatic patients have lower antibody titres than those recovering from severe COVID-19 [42, 43]. A previous positive RT-PCR test for SARS-CoV-2 was reported by four participants in our study, yet only one of these had measurable antibodies by the time the study was performed. All 4 had had their positive RT-PCR more than 5 months prior to serum sample collection. One had experienced no symptoms of disease. The decline in antibody titre over time, especially in asymptomatic individuals, makes seroprevalence studies for epidemiologic purposes difficult to interpret. It also poses a serious threat to any hopes for herd immunity, which is considered a key concept for epidemic control [45]. It further impacts the effectiveness of immunisations if neutralising antibody titres persist only for short periods. However, the role of T cell immunity is increasingly gaining attention. The presence of SARS-CoV-2 specific memory T cells has been observed in seronegative convalescent individuals [46, 47], opening doors to new diagnostic strategies and guiding the development of effective vaccines [48].

### Role of children in the COVID-19 pandemic and in relation to our study findings

An obvious question is, whether healthcare workers in pediatrics are at a lower risk for COVID-19 than their counterparts in adult medicine by virtue of a possible lower infectivity of children.

The role of children in the spread of SARS-CoV-2 remains subject to ongoing research. Dattner et al. estimate that individuals under the age of 20 years are 15% less infectious than those older than 20 years [49]. In a literature review, Goldstein et al. found children to be less susceptible to infection, but evidence confirming a lower infectivity was limited [1].

Transmission from children to other individuals does occur [50] and may [3, 51, 52] or may not [52, 53] lead to outbreaks in schools. In a German study transmission among children in schools and child care facilities was low [54]. Local COVID-19 incidence, outbreak-control strategies including social distancing at school, and early isolation of suspected cases likely play a role in whether or not an outbreak occurs [1].

Additionally, infectivity may differ between younger and older children. Fontanet et al. found that primary school children did not infect their peers, whereas high school children effectively transmitted the virus to others [52]. A South Korean team reconstructed chains of infection and found that the highest transmission among all age groups, including adults, occurred among children aged 10–19, and the lowest occurred in children aged 0–9 [55]. More than two-thirds of children presenting to our hospital are under the age of 10 years, which may be a further protective factor to our staff.

Jones et al., on the other hand, measured viral loads of SARS-CoV-2 in individuals across all age groups and found differences that were more likely due to circumstantial factors, such as timing in the course of the disease, and the authors concluded that viral loads (and, therefore, infectivity) were similar across all age groups. The lower proportion of paediatric index cases among clusters [56], therefore, may initially have been due to infections being brought in from travelling adults during the first phase of the pandemic, and not due to low infectivity of children.

Our study shows that healthcare workers who come into frequent, close contact with children and who use adequate PPE are not at a higher risk of COVID-19 exposure than the general population. However, a final conclusion about the role of children in the spread of the pandemic remains elusive.

The recent emergence of more highly transmissible SARS-CoV-2 variants, such as B.1.1.7, B.1.351, and P.1 [57–59], may alter this picture considerably.

### Self-perceived risk

Perceived personal risk may be an expression of how serious the threat by the COVID-19 pandemic is considered, and of confidence placed in measures applied to limit it. It drives preventive behaviours on the one hand [13, 60, 61], but it also predicts negative mental health outcomes [60, 62]. A nationwide survey in Germany found that 60% of HCWs had concerns regarding their own health [14]. Behrens et al. found that perceived risk among HCWs at a German hospital was considerable while actual seroprevalence was low [15].

Of note, we had asked participants about perceived probability of having already been infected in the past, whereas many other studies ask about perceived risk of a future infection [13, 60, 62]. The positive correlation with previous symptoms of a respiratory illness is, therefore, unsurprising. The negative association with age and the variability among different professions are more difficult to interpret. We controlled for confounding by the measured variables, but we cannot exclude confounding by other factors.

While self-perceived risk in the form of a probability score cannot be directly compared to seroprevalence, we find a definite discrepancy between the two. More than a quarter (*n* = 131) of study participants rated their risk at 50% or higher, whereas only 0.325% (*n* = 2) had a positive antibody test. This probably reflects the uncertainty within a pandemic with a pathogen not well understood at the time, where the general narrative is that healthcare workers are at a particularly high risk of infection. In a cohort that was informed of its serological test results, which were mostly negative, self-perceived risk decreased significantly [15].

Understanding risk perceptions in the general public and among subgroups may ultimately guide effective communication about the pandemic to the public.

### Strengths and limitations

Participation was largely based on voluntary response to generalised announcements and some targeted requests. The overall response was high (70.3%), but response from trainees and service staff was below the overall response rate, and administrative staff, physicians, and allied health staff were represented to a greater extent. However, this is the only hint for non-representative sampling. Almost all children presenting to our hospital are accompanied by an adult; therefore, staff is exposed to both populations almost equally and the measured seroprevalence must be interpreted in this context. As mentioned, antibody titres may decline over time, compromising the epidemiological value of our results. Risk perception could have been related to individual understanding of COVID-19.

In conclusion, SARS-CoV-2 seroprevalence is low among healthcare workers at a large children’s hospital in Germany after the first epidemic wave. Several protective factors may play a role, including low local COVID-19 incidence, use of personal protective equipment, and screening of inpatients. The seroprevalence is far from a level that confers herd immunity. Because the second epidemic wave strikes with greater force than the first, it is important to remember that the above-mentioned preventive measures work and should be continued. The discrepancy between perceived risk of infection and actual seroprevalence may be a reflection of the general narrative that healthcare workers are at a particularly high risk of infection.

## Data Availability

All data will be made available upon request.
